# Hybrid procedure to treat aortic arch aneurysm combined with aortic arch coarctation and left internal carotid artery aneurysm (Case Report)

**DOI:** 10.1186/1749-8090-9-3

**Published:** 2014-01-03

**Authors:** Weimin Zhou, Wei Zhou, Jiehua Qiu, Qingzhong Zeng

**Affiliations:** 1 Department of Vascular Surgery, the second affiliated hospital of Nanchang University, No 1#, Minde Road, Nanchang, China

**Keywords:** Aneurysm, Aortic arch, Stent graft, Hybrid procedure

## Abstract

Aortic arch aneurysm is a rare condition but carries a high risk of rupture. We report one case of aortic arch aneurysm combined with aortic arch coarctation and left internal carotid artery aneurysm, which is extremely rare. Left internal carotid artery aneurysm resection and revascularization, carotid and carotid graft bypass combined with endovascular stent graft and embolization with coils were successfully performed. There were no any complaints and complications at 8 months follow-up. The follow-up CTA demonstrated thrombus formation in the aneurysm lumen, no endoleak and the aortic arch and bypass graft were all patent. We feel that hybrid procedure may be a valuable therapeutic alternative when treating this type of lesion. However, long-term clinical efficacy and safety have yet to be confirmed.

## Background

Aortic arch aneurysm is a rare condition but carries a high risk of rupture. Previous reports that we have identified in English literature included conventional surgical repair, hybrid surgery and embolization of an aortic arch aneurysm with detachable coils [1-5]. Conventional surgical intervention requires a thoracotomy, cardiopulmonary bypass, hypothermic circulatory arrest and aortic cross-clamping, thus remains a surgical challenge with a high rate of mortality (7-17%) and neurologic complication (4-12%) [1,2,6]. We report one case of aortic arch aneurysm combined with aortic arch coarctation and left internal carotid artery (LICA) aneurysm, which is extremely rare. Hybrid procedure was performed successfully, which appears to be a promising minimally invasive approach to manage this rare entity.

## Case presentation

**Figure 1:**
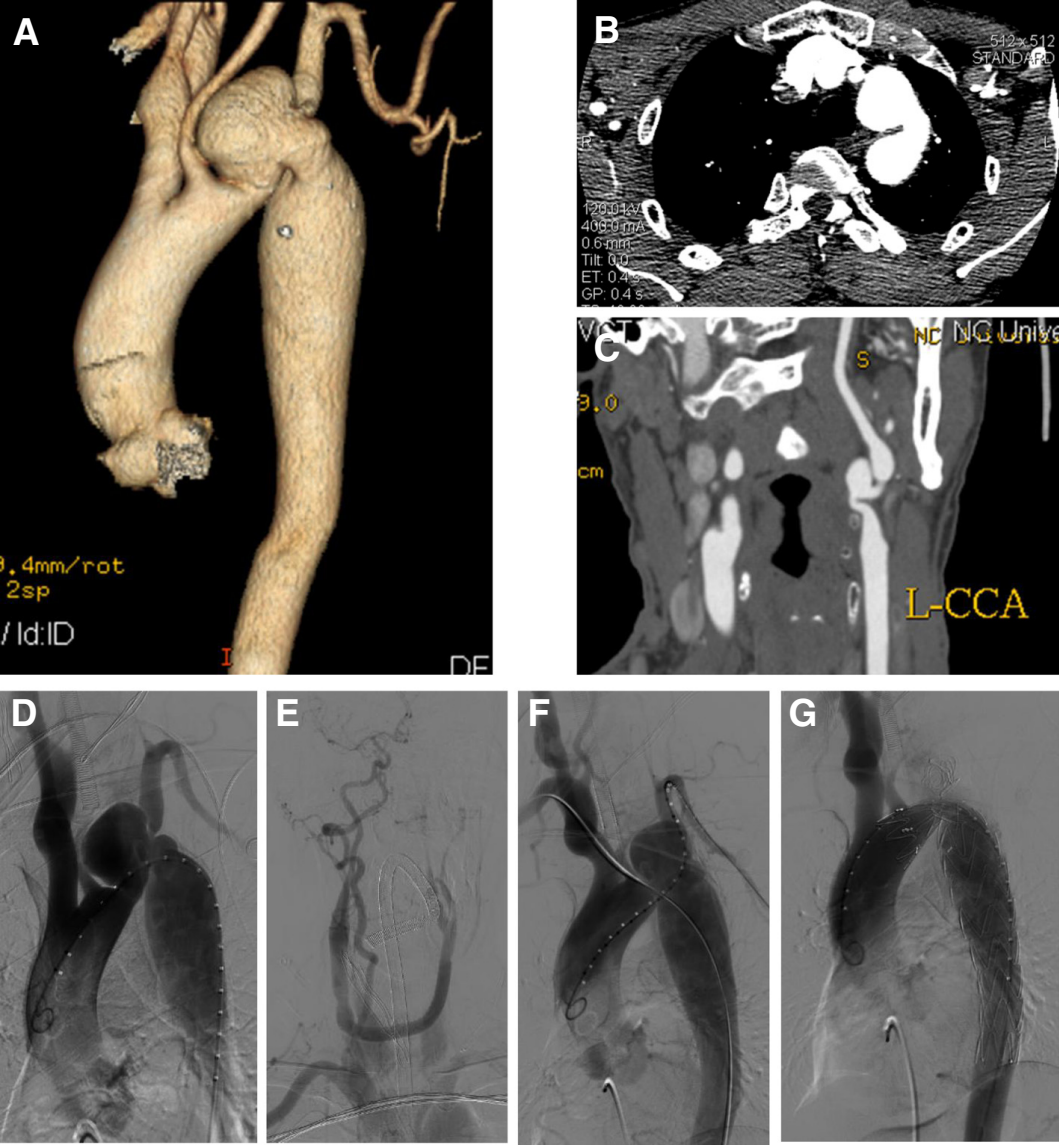
**CTA demonstrated an aortic arch with 50 mm sized saccular aneurysm at 3 mm distal to the LCCA, aortic arch coarctation in the size of 12 mm (A, B), and a LICA aneurysm (C).** DSA was in agreement with the CTA images **(D)** and showed patency of the carotid to carotid bypass **(E)**; Brachial-femoral stretch guidewire technique was performed **(F)**; The completion aortogram revealed no evidence of endoleak; disappearance of the aortic arch aneurysm lumen **(G)**.

**Figure 2:**
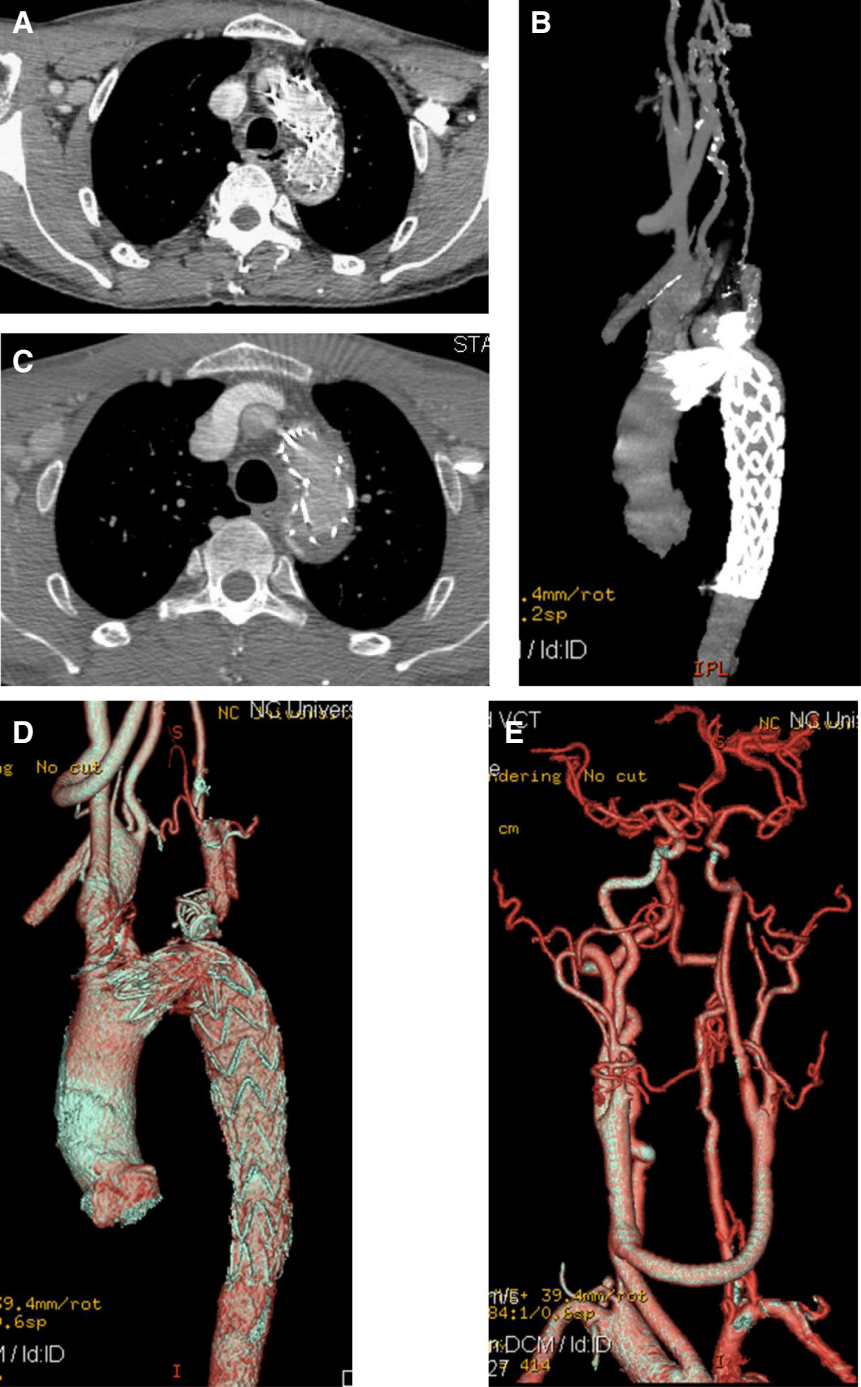
**CTA demonstrated aortic arch aneurysm sac with thrombosis and a small endoleak, and patent carotid to carotid bypass in one week follow-up (A, B).** Postoperative CTA demonstrated thrombosis in the aneurysm lumen, no endoleak and the aortic arch and the carotid to carotid bypass were all patent except very mild stenosis of LICA at 8-month follow-up **(C, D, E)**.

A 56-year-old Chinese male patient was admitted to our center with left sided chest pain for one year. A computer tomography angiography (CTA) demonstrated an aortic arch aneurysm, aortic arch coarctation and LICA aneurysm. The CTA also showed a 50 mm sized saccular aneurysm 3 mm distal to the left common carotid artery (LCCA), the left subclavian artery (LSA) arose from the aortic arch aneurysm and the size of aortic arch coarctation was 12 mm (Figure 1A-C). The patient refused to undergo conventional surgical repairment, however, endovascular therapy alone would not treat all the lesions of this patient. Therefore, we decided to perform a hybrid procedure that consisted of a LICA aneurysm resection and revascularization, carotid to carotid bypass operation and the placement of stent graft in the aortic arch aneurysm to save the LCCA, coils embolization of the aortic arch aneurysm and aortic arch coarctation dilatation. The procedure was performed under general endotracheal anesthesia with full hemodynamic monitoring one week later after admission. We performed LICA aneurysm resection and the carotid to carotid bypass operation connecting from the right carotid artery to the left carotid artery in advance for revascularization of the post-stent grafting in the hybrid room one step. The right femoral artery was exposed through a groin oblique incision and cannulated with a 10-French catheter sheath from which an angiographic mark catheter was put into the ascending aortic artery via a 0.035 inch guidewire. A digital subtraction angiography (DSA) was performed which was in agreement with the CTA images. It also confirmed the patency of the carotid to carotid bypass graft and showed dominant right vertebral artery (Figure 1D, E). The “C” arm was locked at left anterior oblique (LAO) 50° projection to thoroughly unfold the aortic arch that was cannulated with a 21-French delivery introducer sheath via a 0.035 inch Lunderquist super stiff guidewire (COOK, USA) through the right femoral artery. A thoracic endoprosthesis stent graft (TF3232C200X, VALIANT®, Medtronic Inc., USA) was deployed. Unfortunately, due to the kink of the aortic arch, the stent graft could not be deployed. Therefore, the right brachial artery was punctured and cannulated with a 6-French catheter sheath from which a snare (AMPLATZ GOOSE NECK, ev3 Inc., USA) was inserted into the ascending aortic artery to capture the Lunderquist super stiff guidewire (brachial-femoral stretch guidewire) (Figure 1F). The stent graft was accurately deployed through the stretch wire, and a balloon 20 mm in diameter (Atlas, BARD Inc., USA) was inserted to dilate the coarctation lesion at the aortic arch. The patient had immediate relief of coarctation, with residual gradient decreasing from 38 mmHg to 7 mmHg. Then the left brachial artery was punctured and cannulated with a 6-French catheter sheath from which a 5 F vertebral catheter was inserted into the aortic arch aneurysm via a 0.035 inch guidewire, and four 15 mm coils were placed into the aortic arch aneurysm sac. The completion aortogram revealed no evidence of endoleak; disappearance of the aortic arch aneurysm lumen and patency of the carotid to carotid bypass (Figure 1G). Systemic heparinization was administrated during the procedure. His postoperative course was uneventful and discharged on postoperative day 7 with a follow-up CTA demonstrating aortic arch aneurysm sac thrombosis with a little type I endoleak and patent carotid to carotid bypass (Figure 2A, B). As of 8 months after the hybrid procedure, the patient is well without any complaints and complications. A follow-up CTA demonstrated thrombosis in the aneurysm lumen, no endoleak. The aortic arch and the carotid to carotid bypass were both patent except very mild stenosis of LICA and aortic arch (Figure 2C, D, E).

## Discussion

Standard treatment for aortic arch aneurysm is open surgery, due to its good long term results [7,8]. But surgical repair of the aorta requires thoracotomy, cardiopulmonary bypass, hypothermic circulatory arrest, aortic crossclamping and systemic heparinization. Conventional surgical repair for aortic arch pathology carries a high mortality and morbidity, with a particularly significant incidence of neurologic injury [1,6,9]. Therefore they were gradually replaced by endovascular treatment due to the complexity of the surgery, surgical trauma and high associated mortality rate. Endovascular treatment is less invasive and is associated with lower morbidity and mortality [3-5]. Since endovascular procedure does not require thoracotomy, circulatory assistance is not necessary and significant haemorrhages are less likely. What is more, endovascular intervention does not need aortic cross-clamping as such the risk of cerebral, spinal cord and visceral ischemia was decreased. Due to the lower morbidity and mortality rates, thoracic endovascular aortic repair (TEVAR) is considered an acceptable alternative to open surgical repair for patients with various types of aortic diseases. With the successful experience including ours, using of a fenestrated or branched stent graft and double chimneys, which is able to preserve perfusion of the supra-aortic arch vessels, could be one of the alternative approaches [10,11]. However, simple application of TEVAR to treat aortic arch aneurysm combined with aortic arch coarctation and LICA aneurysm such as our case may cause cerebral ischemia and infarction. In this case, the aortic arch aneurysm located at the greater curvature, combining with aortic arch coarctation and LICA aneurysm, and the LSA was arising from the aortic arch aneurysm. Endovascular therapy could not repair all the lesions; however, conventional open surgery would be complicated and have high associated mortality rate, so hybrid procedure was a good alternative approach. Because of the dominant right vertebral artery, we could occlude the LSA but without causing cerebral ischemia or cerebral infarction. But if the patient presents with left arm ischemia, a subclavian to subclavian artery or axillary to axillary artery graft bypass could be performed. Although there was a very mild stenosis of LICA and aortic arch at 8-month postoperatively, but the patient does well without any complaints and complications. Our successful experience in this patient suggests that the combined endovascular and surgical treatment seems to be a valuable therapeutic alternative when treating this type of aortic arch lesion with advantages of performing less aggressive surgery and avoiding aortic cross-clamping, circulatory assistance and high dose heparinization. But long-term follow-up of a larger number of patients is needed to assess and confirm these favorite results in order to promote these approaches.

## Conclusion

Our case suggests that hybrid procedure in treating aortic arch aneurysm combined with aortic arch coarctation and LICA aneurysm is a better choice. The use of combined open and endovascular repair in the treatment of aortic arch pathology appears safe and effective at perioperative, postoperative and early midterm follow-up and offers several advantages over conventional surgical repair, including the potential to offer therapy to patients who are not candidates for open repair.

## Consent

Written informed consent was obtained from the patient for publication of this case report and any accompanying images. A copy of the written consent is available for review by the Editor-in-Chief of this journal.

## Abbreviations

CTA: Computer tomography angiography; LICA: Left internal carotid artery; LCCA: Left common carotid artery; LSA: Left subclavian artery; DSA: Digital subtraction angiography; LAO: Left anterior oblique; TEVAR: Thoracic endovascular aortic repair.

## Competing interests

The authors declare that they have no competing interests.

## Authors’ contributions

WnZ analyzed and interpreted the patient data. WiZ, JQ and QZ were the major participants of the operation and had participated in its design and coordination. All authors have read and approved the final manuscript.
